# Stereotactic body radiation therapy for clinically diagnosed early‐stage non‐small cell lung cancer: Importance of accurate CT interpretation by experts

**DOI:** 10.1002/pro6.1220

**Published:** 2024-03-10

**Authors:** Daisuke Nakamura, Koichi Honda, Takuya Yamazaki, Hideyuki Hayashi, Shin Tsutsui, Aya Fukushima, Sumihisa Honda, Masataka Uetani, Kazuto Ashizawa

**Affiliations:** ^1^ Department of Clinical Oncology Nagasaki University Graduate School of Biomedical Sciences Nagasaki Japan; ^2^ Department of Radiology NHO Nagasaki Medical Center Omura Japan; ^3^ Department of Radiological Sciences Nagasaki University Graduate School of Biomedical Sciences Nagasaki Japan; ^4^ Department of Radiology JCHO Isahaya General Hospital Isahaya Japan; ^5^ Department of Public Health & Nursing Nagasaki University Graduate School of Biomedical Sciences Nagasaki Japan

**Keywords:** lung neopasms, oncologic imaging, radiation oncology imaging

## Abstract

**Introduction:**

This study evaluated the clinical outcomes of stereotactic body radiotherapy (SBRT) for both pathologically diagnosed (PD) and clinically diagnosed (CD) early‐stage non‐small cell lung cancer (NSCLC) and explored the significance of accurate expert computed tomography (CT) interpretation.

**Methods:**

We retrospectively analyzed 95 patients with early‐stage NSCLC who received SBRT at our institution. Patients were classified into CD and PD groups. Two chest radiologists retrospectively interpreted the pre‐SBRT CT images to determine the tumor subtype and probability of malignancy (PM). Clinical findings, CT features, and treatment outcomes were compared between the two groups. The survival rate of the CD group was analyzed separately according to the PM grade.

**Results:**

Median overall survival for the CD and PD groups was 6.0 and 5.4 years (*P* = 0.57), respectively. Median cause‐specific survival was 10.2 years in the CD group and not reached in the PD group (*P* = 0.76). In the CD group, lung cancer mortality was lower in the low PM group (25% [1 of 4]) than in the high PM group (47.4% [9 of 19]).

**Conclusion:**

It may be desirable to evaluate the PM of the nodule using expert CT interpretation to decide whether SBRT is indicated in CD early‐stage NSCLC.

## Graphical abstract

The present study investigated the clinical outcomes of stereotactic body radiotherapy (SBRT) for pathologically diagnosed (PD) and clinically diagnosed (CD) early‐stage non‐small cell lung cancer and examined the importance of accurate CT interpretations by experts. Overall survival and cause‐specific survival curves showed no significant difference between the PD and CD groups. (a, b). The CD group was divided into the low probability of malignant (PM) group and the high PM group by CT interpretation. Although there was no significant difference in the OS and CSS curves of the high and low PM groups (c), lung cancer mortality was lower in the low PM group (25% [1 of 4]) that in the high PM group (47.4% [9 of 19]).

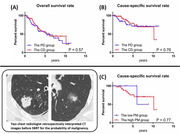

## 1 INTRODUCTION

The standard treatment for early‐stage lung cancer is surgical resection.^1^ However, recent studies showed that the clinical outcomes of patients with early‐stage non‐small cell lung cancer (NSCLC) who received stereotactic body radiotherapy (SBRT) were similar to those of surgery.^2^ SBRT is recommended as an alternative therapy for medically inoperable patients or those refusing surgery.^3^

Although a pathological diagnosis (PD) is essential for the definitive diagnosis of lung cancer, clinicians are sometimes faced with managing lung nodules in patients in whom a diagnostic biopsy is not possible because of reduced lung function or severe complications. Ground‐glass nodules (GGN) on computed tomography (CT) can be used to distinguish between benign and malignant lesions. Therefore, CT findings of lung cancer corroborate PD. Moreover, ^18^ Fluorine‐Fluorodeoxyglucose Positron Emission Tomography(^18^ F‐FDG PET) shows high diagnostic accuracy for the differential diagnosis of benign versus malignant lesions.^4^ The number of NSCLC patients treated by SBRT without PD is increasing.^5^, ^6^ However, some of the lesions treated by SBRT without PD may be benign.^6^, ^7^, ^8^

The present study investigated the clinical outcomes of patients with both PD and clinically diagnosed (CD) early‐stage NSCLC, who received SBRT and examined the importance of accurate CT interpretations by experts.

## 2 MATERIALS AND METHODS

### 2.1 Patients characteristics

This study included 122 consecutive patients treated with SBRT for lung tumors, between 2008 and 2014, at Nagasaki University Hospital. Chest CT and PET‐CT were performed in all patients. Of the 122 patients 27 diagnosed with lung metastases, small‐cell carcinoma, or positive lymph nodes were excluded. The remaining 95 patients with clinical T1‐2N0M0 NSCLC were included in the analysis. All 95 patients were determined ineligible for surgery by a thoracic tumor board consisting of radiation oncologists, chest radiologists, pulmonologists, and thoracic surgeons for reasons such as poor pulmonary function, comorbidities, or refusal to undergo surgery. Patients with PD NSCLC were included in “the PD group”, and patients with CD NSCLC without PD as “the CD group”. There were 48 and 47 patients in the PD and CD groups, respectively. In the CD group, a pathological diagnosis was not obtained for various reasons. In 24 patients with suspected lung cancer, a definitive diagnosis was not received despite undergoing a bronchoscopy, and 19 were unable to undergo bronchoscopic or CT‐guided biopsy due to poor lung function or severe complications such as interstitial pneumonia, chronic heart failure, and pulmonary hypertension, and 4 patients deferred these examinations. Indication of SBRT in the CD group was decided by the chest tumor board, which also considered the probability of malignancy based on CT images of pulmonary lesions.

### 2.2 Imaging analysis

Two chest radiologists with 19 and 13 years of experience, affiliated with other facilities and not informed of the clinical findings, retrospectively interpreted the CT images of the lung lesions after SBRT. Chest CT images were obtained with a 1 mm slice thickness using multidetector row CT. The window width for the interpretation of lung lesions was 1600 HU, and the window center was ‐600 HU. Lung lesions were classified into three subtypes: solid, part‐solid, and pure GGN. They also classified the probability of malignancy (PM) of the lesions into five grades (1, definitely benign; 2, probably benign; 3, possibly malignant; 4, probably malignant; and 5, definitely malignant)

### 2.3 Treatment

Patients were treated using the PRIMUS and Novalis Tx accelerators, which were equipped with in‐room CT and cone‐beam CT, respectively. Both accelerators were equipped with a micro‐multileaf collimator (MLC), an ExacTrac system, and a Robotic Tilt Motion mounted on the Exact Couch top. The patients were immobilized using a thermoplastic shell with infrared markers. Breath‐hold CT images were acquired four times with a slice thickness of 2 mm. Treatment plans were created using Pinnacle or Eclipse, version 8.6. Breath‐hold CT images were fused with these treatment planning systems.

Gross tumor volume (GTV) was defined as the volume of the tumor including the spiculation. The clinical target volume (CTV) was the same as that of the GTV. The planning target volume (PTV) was defined as the CTV plus an 8–10 mm margin in all directions. SBRT was performed using multiple static beams of 6‐MV photons. MLC margins of 5 mm were added to the PTV. SBRT was administered to the isocenter with breath‐holding. The SBRT dose was selected at the discretion of the attending physician and was based on the size and location of the tumor. Peripheral tumors received 48 Gy in 4 fractions, while central tumors received 50 or 60 Gy in 10 fractions.

### 2.4 Follow‐up and statistical analysis

Patients were routinely followed up and underwent CT every 3–6 months. Continuous variables were compared between the PD and CD groups using the Mann‐Whitney test, and the same was used for the T category (UICC 8^th^) as an ordinal variable. Categorical variables were compared using the chi‐squared test. The Kaplan–Meier method and log‐rank test were used to examine overall survival (OS) and cause‐specific survival (CSS) from the initiation of SBRT. Hazard ratios were assessed using Cox regression analysis. The weighted kappa statistic was used to evaluate the reproducibility of PM of the nodules between the two chest radiologists. A *P*‐value < 0.05 was considered a significant difference. All statistical analyses, except the weighted kappa statistics, were conducted using GraphPad Prism 6 and JMP pro17.

## 3 RESULTS

**FIGURE 1:**
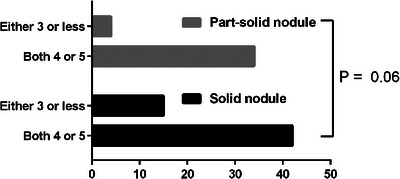
Grade of the probability of malignancy.

Table 1 presents the patient characteristics. The PD group had a larger primary tumor size, resulting in a significant difference in the proportion of T category tumors (*P* = 0.02). The CD group had a higher rate of interstitial pneumonia (*P* = 0.02). No significant differences were observedin the other factors between the two groups. Additionally, there was no significant difference in the ratio of three nodule subtypes including solid nodule (PD group:32[66.7%] vs. CD group:25[53.2]),  part‐solid nodule (PD group:16[33.3] vs. CD group:22[46.8]) and pure GGN (0) assessed by the two chest radiologists in the CT interpretations between the two groups (*P* = 0.21). The kappa value indicated a high concordance rate for the PM of nodules evaluated by both chest radiologists (*κ* = 0.95). None of the nodules were classified as PM grade 1 or 2. The proportion of PM grade 3 nodules in part‐solid nodules was lower than in solid nodules; however, the difference was not statistically significant (*P* = 0.06) (Figure 1).

**TABLE 1:** Patient characteristics.

	The PD group (n = 48)	The CD group (n = 47)	*P*‐value
Median age (IQR) (years)	76 (71–81)	76 (72–80)	0.74
Male sex, *n* (%)	34 (70.8)	32 (68.1)	0.83
Histological type, *n* (%)			
Adenocarcinoma	28 (58.3)		
Squamous cell carcinoma	15 (31.3)		
Carcinoma	5 (10.4)	Not available	
T category, *n* (%)			
T1a	2 (4.2)	6 (12.8)	
T1b	13 (27.1)	18 (38.3)	
T1c	18 (37.5)	16 (34.0)	
T2a	11 (22.9)	5 (10.6)	
T2b	4 (8.3)	2 (4.3)	0.02*
Median FEV1 (IQR) (ml)	1565 (955–2048)	1730 (1300–2005)	0.46
Current and past smoker, *n* (%)	35 (72.9)	36 (76.6)	0.68
Interstitial pneumonitis, *n* (%)	0 (0)	4 (8.5)	0.02*
Previous history of cancer, *n* (%)	24 (50)	26 (55.3)	0.68
Prescribed dose, *n* (%)			
48 Gy in 4 fractions	30 (62.5)	31 (66.0)	
50 Gy in 10 fractions	2 (4.2)	3 (6.4)	
60 Gy in 10 fractions	16 (33.3)	13 (27.6)	0.67

Abbreviations: CD, clinical diagnosis; FEV1, forced expiratory volume in 1 s; IQR, interquartile range; PD, pathological diagnosis.

**FIGURE 2:**
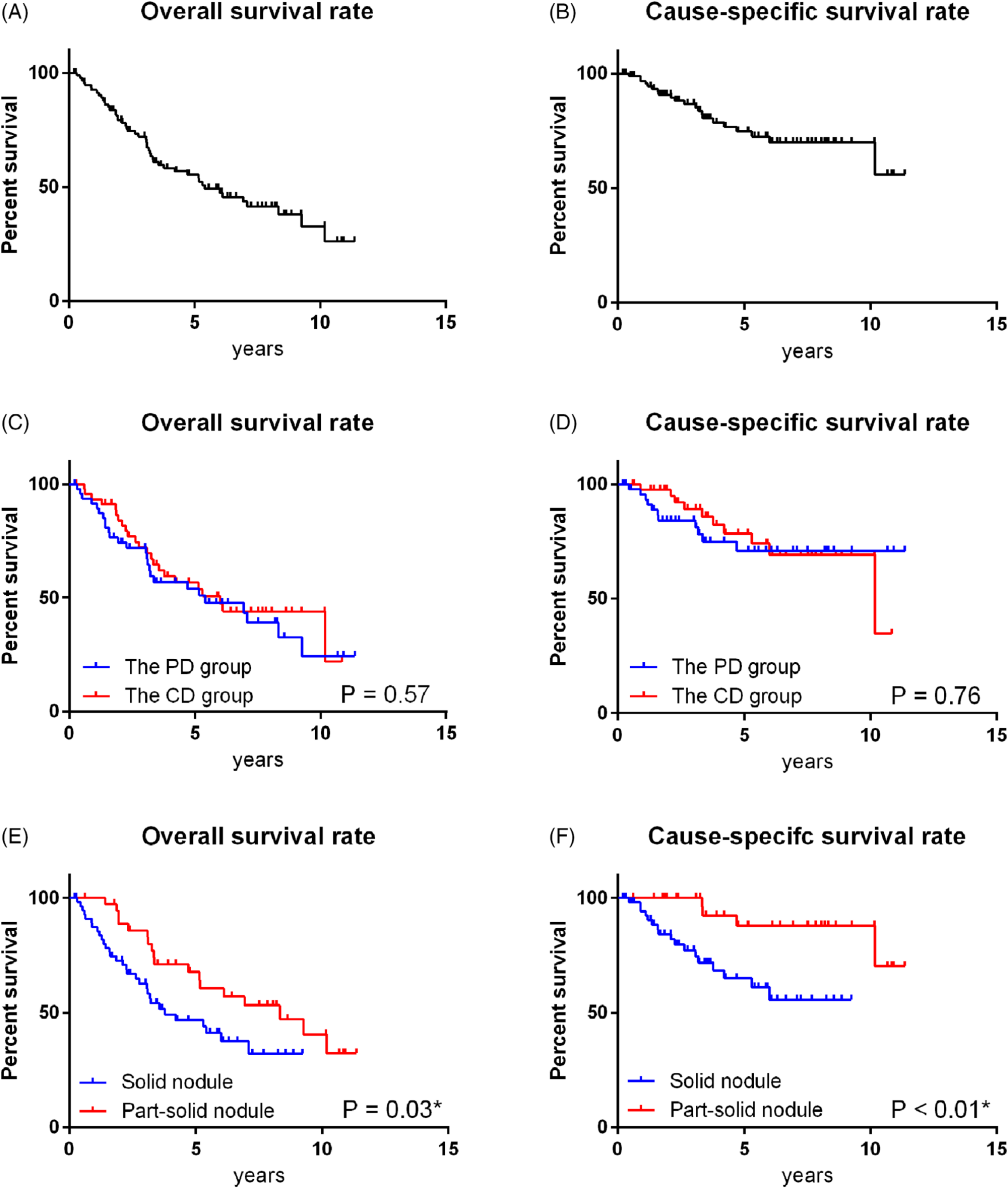
Kaplan‐Meier curves: overall survival rate of all patients (A), cause‐specific survival rate of all patients (B), overall survival rates of the PD and CD groups (C), cause‐specific survival rates of the PD and CD groups (D), overall survival rates of part‐solid nodules and solid nodules (E), and cause‐specific survival rates of part‐solid nodules and solid nodules (F). CD,clinically diagnosed; PD, pathologically diagnosed.

The median follow‐up period for surviving patients was 5.8 years. Of the total patients, 21 died of the primary disease and 28 from other diseases. The median OS was 6.0 years and 5.4 years in the CD and PD groups, respectively, with no significant difference (*P* = 0.57). The median OS was significantly longer for part‐solid nodules (8.3 years) than for solid nodules (3.8 years) (*P* = 0.03). The median CSS was 10.2 years in the CD group, but was not reached in the PD group, with no significant difference (*P* = 0.76). The CSS curves showed better survival for part‐solid nodules than for solid nodules (*P* < 0.01) (Figure 2).

**FIGURE 3:**
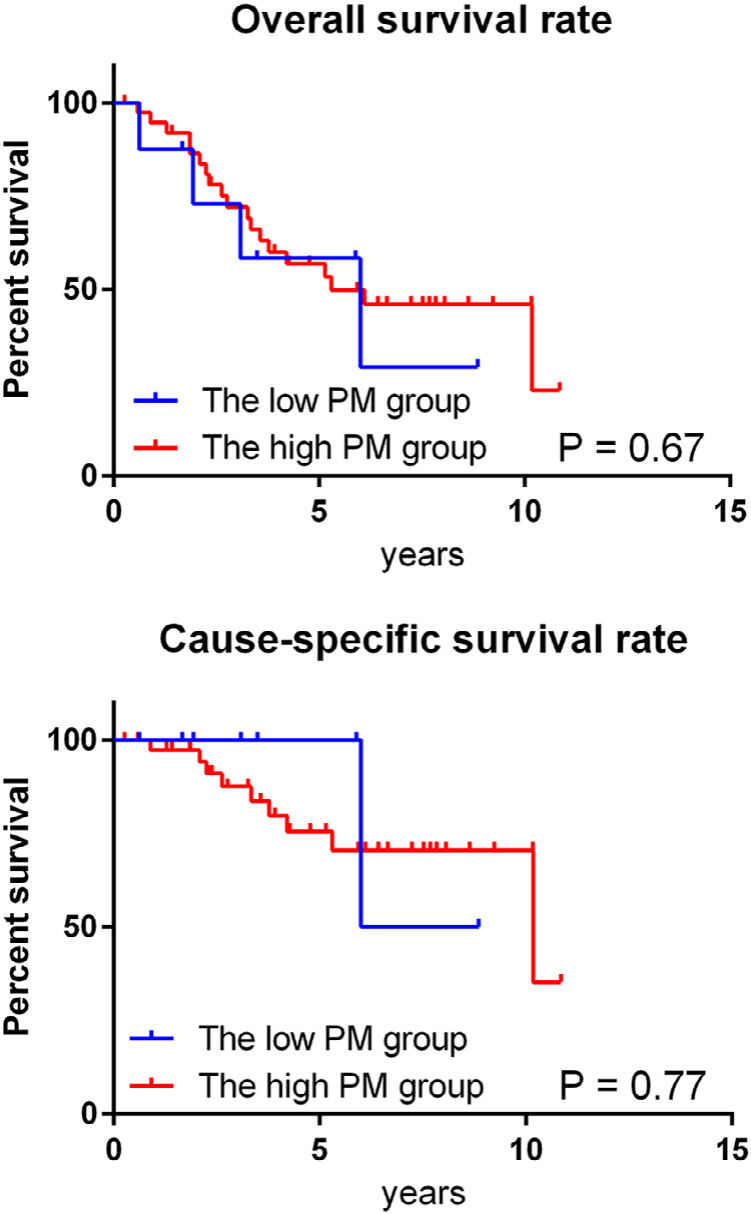
The survival curve of the clinically diagnosed group by PM levels. PM, probability of malignancy.

The treatment outcomes in the CD group were analyzed separately based on the PM grades assigned by the chest radiologists. The low PM group included cases in which either of the two radiologists assigned a grade of 3, whereas the high PM group included cases in which both radiologists assigned a grade of 4 or higher. Although there were no significant differences in the OS and CSS curves between the two groups (Figure 3), lung cancer mortality was lower in the low‐PM group (25% [1 of 4]) than in the high‐PM group (47.4% [9 of 19]).

Cox proportional hazards regression analysis of survival hazard ratios in the univariate models was performed (Table 2). We also used a multivariable Cox regression model that included a significant *P*‐value (*P* < 0.05) in the univariable models (sex, tumor subtype, and smoking history) and estimated hazard ratios. None of these three factors proved to be significant prognostic factors in the multivariate analysis.

**TABLE 2:** Prognostic factors by Cox proportional hazards models.

Univariate analyses		
Factors	Hazard ratio (95% CI)	*P*‐value
Age (years old)		
Under 70 / 70–79	0.86 (0.40 – 1.86)	0.71
Under 70 / Over 80	0.77 (0.35 – 1.67)	0.51
70–79 / Over 80	0.89 (0.48 – 1.67)	0.72
Sex (male / female)	5.23 (2.31 – 11.9)	<0.01*
Pathological confirmation (PD / CD)	1.14 (0.65 – 2.03)	0.64
T category (T1 / T2)	0.75 (0.37 – 1.52)	0.42
Interstitial pneumonia (yes / no)	2.11 (0.65 – 6.9)	0.22
Previous history of cancer (yes / no)	1.00 (0.57 – 1.75)	0.99
Tumor subtype (solid nodule / part‐solid nodule)	1.92 (1.04 – 3.54)	0.04*
FEV1 (ml)		
Under 1000 / 1000–1999	0.95 (0.40 – 2.27)	0.92
Under 1000 / Over 2000	0.61 (0.25 – 1.45)	0.26
1000–1999 / Over 2000	0.63 (0.34 – 1.19)	0.16
Smoking history (yes / no)	5.01 (2.08 – 12.0)	< 0.01*
Prescribed dose (48 Gy in 4 fractions / 50 and 60 Gy in 10 fractions)	1.22 (0.65 – 2.29)	0.53

Multivariate analysis		
Factors	Hazard ratio (95% CI)	*P*‐value
Sex (male / female)	3.07 (0.73 – 12.9)	0.13
Tumor subtype (solid nodule / part‐solid nodule)	1.57 (0.85 – 2.93)	0.15
Smoking history (yes / no)	1.77 (0.38 – 8.17)	0.46

Abbreviations: CD, clinical diagnosis; FEV1, forced expiratory volume in 1 s; PD, pathological diagnosis.

Six patients experienced adverse events. Four patients experienced grade 3 radiation pneumonitis, and one patient experienced grade 4 dyspnea. Unfortunately, one patient in the CD group with idiopathic pulmonary fibrosis and a KL‐6 level of 605 U/ml died of radiation pneumonitis 7 months after SBRT.

## 4 DISCUSSION

A systematic review and meta‐analysis of 43 studies involving 47 cohorts of NSCLC patients undergoing SBRT were conducted, five of which directly compared oncologic outcomes between clinical and biopsy‐proven NSCLC. The results revealed that patients who received SBRT for early‐stage NSCLC had better CSS in the CD group demonstrating differences in oncological outcomes. These findings emphasize the importance of pathological proof of malignancy.^8^ However, four comparative studies^9^, ^10^, ^11^, ^12^ found no difference in CSS, and one remaining study, an analysis of the U.S. SEER program with the largest number of cases,^6^ did not report the percentage of patients who underwent CT or PETCT, or the criteria for clinical diagnosis. In contrast, in this study, there was no significant difference in OS or CSS between the CD and PD groups, nor was there a significant difference in PM based on CT interpretation between the two groups. The indication criteria for SBRT in the CD group at our hospital may have prevented the difference in CSS rates between the CD and PD groups by reducing benign tumor contamination as much as possible.

The percentage of patients in the CD group in our hospital was 50%, which is higher than that in other reports.^6^, ^9^, ^10^, ^11^, ^12^ The reasons why a PD was not reached in the CD group have already been described. The Charlson Comorbidity Index^13^ with a median score of 7 (range 5–12) in the CD group, was high, raising concerns about complications associated with invasive examinations. We believe that this is one of the reasons the proportion of the CD group was high in the present study. However, based on the OS and CSS results in this study, we believe that our treatment policy of applying SBRT without additional examinations in the CD group was justified.

The kappa value of the PM grade was high, indicating that the high reproducibility of PM evaluation by CT interpretation. These results indicate that patients with benign nodules can be excluded as much as possible by determining the indications for SBRT on the cancer board. Furthermore, when evaluating the treatment results in the CD group according to the PM grade by CT interpretation, there was no difference in the survival results between the high and low PM groups. However, the rate of cancer deaths was lower in the low PM group. This indicates that PM evaluation by experts may be important when applying SBRT to the CD group.

Although not significantly different, the tumor subtypes in the CD group had a higher proportion of part‐solid nodules than those in the PD group. Part‐solid nodules are reported to have a higher association with adenocarcinoma.^14^ Further, part‐solid nodules exhibited a comparatively higher frequency of high PM grades than solid nodules. This suggests that CT interpretation by experts may help avoid unnecessary lung biopsies for the definitive diagnosis of part‐solid nodules in cases where biopsy poses a risk.

Previous studies have reported that the proportion of solid components in lung nodules contributes to the prognosis of patients after surgery.^15^, ^16^ In this study, OS and CSS were significantly longer for part‐solid nodules than for solid nodules. Moreover, part‐solid nodules with small tumor diameters indicated a very good prognosis, but may not serve as prognostic factors for elderly patients with shorter life expectancy.

The mean FEV1 of the patients in this study exceeds 1.5 L, and surgery was indicated in some cases. Previously, our hospital was the only facility in the neighborhood to offer SBRT. Consequently, patients who refused surgery and preferred SBRT, even those with good respiratory function, were more likely to visit our hospital. While our hospital actively performs surgery for patients with early‐stage NSCLC, even those with advanced age and low lung function, studies suggest that the outcomes were not much different from SBRT, indicating the appropriateness of choosing SBRT over surgery.^17^ Another report has shown that SBRT is non‐inferior compared to video‐assisted thoracoscopic surgical lobectomy with mediastinal lymph node dissection.^18^ However, there was no difference in CSS between thoracoscopic sub‐lobar resection and SBRT for small tumors of 2 cm or less. Nevertheless, it was reported that the CSS of thoracoscopic resection was significantly superior to that of SBRT when the tumor diameter was large.^19^ Therefore, SBRT should be carefully indicated for large tumors.

In this study, no significant prognostic factors were identified using multivariate analysis. In contrast, the prognosis of patients with surgically resected NSCLC can be predicted on the basis of age, sex, histological type, stage at diagnosis, and additional treatment. Previous studies have reported that smoking history before the diagnosis of lung cancer also influences the prognosis of patients.^20^ In addition, a significant increase in OS was achieved by smoking cessation after SBRT for early‐stage NSCLC.^21^ Therefore, efforts to encourage smoking cessation by all patients after treatment are considered important.

SBRT is a minimally invasive treatment option. While it is desirable to perform SBRT after PD, using a bronchoscope to obtain samples is an invasive procedure. Owing to the risk of complications, SBRT may be more appropriate for CD rather than PD lung cancer patients with low lung function or comorbidities. Regarding adverse events, in RTOG 0236, grade 3–4 adverse events were observed in 28% of patients, and there were no treatment‐related deaths.^22^ In JCOG 0403, grade 3 radiation pneumonitis developed in 8.8% of patients and grade 4 dyspnea in 1.2%.^23^ Based on these findings, it may be necessary to carefully apply the selection criteria, especially for patients with low lung function, when considering SBRT.

This study has several limitations. This was a retrospective analysis of a limited number of patients at a single institution. Furthermore, although the present study mainly included elderly patients with low lung function, some older patients with low lung function were only followed up without treatment because they were deemed untreatable at our institution. Therefore, there was a selection bias for SBRT.

In conclusion, the outcomes of SBRT for early‐stage NSCLC in the CD and PD groups were similar. Although SBRT is desirable for patients with early‐stage PD NSCLC, it may also be applied to patients with CD NSCLC who have poor lung function or comorbidities and cannot undergo biopsy or surgical excision. Accurate interpretation of the CT findings of lung lesions by experienced chest radiologists may be essential when considering the indication of SBRT for early‐stage CD NSCLC and avoiding unnecessary biopsy of part‐solid nodules.

## CONFLICT OF INTEREST STATEMENT

The authors declare no potential conflicts of interest.

## ETHICAL STATEMENT

This study was approved by the Clinical Research Ethics Committee of the Nagasaki University Hospital. All procedures were performed in this study in accordance with the 1964 Declaration of Helsinki and its later amendments or comparable ethical standards.

## PATIENT CONSENT TO PARTICIPATE

All patients were enrolled in the study only after providing full consent.
